# Effects of Graded Levels of Mimosa (*Acacia mearnsii*) Tannin Purified with Organic Solvents on Gas, Methane, and In Vitro Organic Matter Digestibility of *Eragrostis curvula* Hay

**DOI:** 10.3390/ani12050562

**Published:** 2022-02-23

**Authors:** Shehu Lurwanu Ibrahim, Abubeker Hassen

**Affiliations:** Department of Animal Science, University of Pretoria, Hatfield, Pretoria 0028, South Africa; abumubarak480@gmail.com

**Keywords:** digestibility, ethyl acetate, inclusion level, methane, mimosa tannin, pentanol, purification, Soxhlet

## Abstract

**Simple Summary:**

Enteric methane emission serves as one of the major contributors to global warming, and is responsible for the loss of an appreciable amount of dietary energy. Utilization of Mimosa tannin in reducing methane yield in ruminants is well documented. However, its supplementation in large amounts affects rumen fermentation and digestibility, attributed to the presence of a high number of hydrolysable tannins and other non-tannin substances, which poison rumen microorganisms. Purification might remove the toxic compounds, thereby decreasing the methane without affecting the digestibility of the diet. In this study, mimosa tannin was purified with organic solvents (ethyl acetate and pentanol) using the Soxhlet extraction method, and the influence of different concentrations (10, 20, 30, and 40 g/kg DM) of the purified tannins on gas, methane, and in vitro organic matter digestibility of *Eragrostis curvula* hay were evaluated in comparison with the same levels of unpurified tannin. The addition of lower levels of ethyl acetate and pentanol purified mimosa tannin extracts reduced in vitro gas and methane production without adverse effects on dry matter digestibility.

**Abstract:**

The higher contribution of methane (CH_4_) to global anthropogenic potential is a cause of concern to livestock producers. Mimosa tannin gained recent acceptance as an additive for enteric CH_4_ mitigation. However, rumen fermentation and digestibility are compromised when large quantities of tannins are supplemented due to the presence of hydrolysable tannin and other non-tannin molecules in mimosa extract, which are toxic to animals. Purification could eliminate the toxins, and thus, reduce the CH_4_ yield without negative effects on rumen microbial activities and organic matter degradation. The Soxhlet extraction method was used to purify the tannin using organic solvents (ethyl acetate and pentanol). The unpurified, ethyl acetate purified, and pentanol purified tannins at the dosages of 10, 20, 30, and 40 g/kg DM of substrate (*Eragrostis curvula* hay) were evaluated for gas, CH_4_, and in vitro organic matter digestibility (IVOMD) in comparison with substrate alone. Gas kinetics were tested using a simple exponential model with lag. The results showed that compared with control, gas, CH_4_, IVOMD, CH_4_/gas, CH_4_/IVOMD, gas/IVOMD, asymptotic gas volume (v), and rate of gas production (k) decreased (*p* < 0.01) linearly with the increase in the inclusion levels of all tannin extracts. Also, ethyl acetate purified and pentanol purified tannin extracts reduced gas and CH_4_ at lower dosage (30 g/kg DM) compared to unpurified tannin extract at a higher level (40 g/kg). Pentanol purified tannin was more effective at lower dosage (20 g/kg DM) in terms of CH_4_/gas and CH_4_/IVOMD. It was concluded that the purification of mimosa tannin with ethyl acetate and pentanol reduced potential gas production and CH_4_ without much reduction in substrate digestibility when up to 30 g/kg DM of feed was used. Lower inclusion levels of ethyl acetate and pentanol purified extracts could give a similar result with a higher dosage of unpurified tannin.

## 1. Introduction

Recent research in ruminant nutrition gave more emphasis to enteric methane (CH_4_) abatement strategies, because of its global warming potential, and the high impact it has on the animal industry. Enteric emissions account for about 39% of the agricultural contribution of global CH_4_ yield per annum [1], and are responsible for the loss of up to 12% of animal energy intake [2]. Several approaches were adopted to moderate CH_4_ production by ruminants, which include animal breeding; improved livestock production; feeding concentrates; antibiotics; and plant secondary compounds, particularly tannins and saponins [3]. However, the use of condensed tannins received more recent attention due to their availability, safety, and efficiency [4].

Mimosa (*Acacia mearnsii*) is regarded as a highly invasive and widely spread tannin-rich legume; extending an area of over 2.5 million hectares [5], in addition to more than 130,000 hectares of commercial plantation [6]. Earlier research showed that mimosa tannin has the potential to reduce enteric CH_4_ and ammonia nitrogen (NH_3_N) production, but with some negative effects on rumen fermentation and digestibility when supplemented above 50 g kg^−1^ DM [7,8,9], attributed to the high amount of hydrolysable tannins, which are regarded as toxic to animals. For instance, when mimosa tannin was included at ≥50 g/kg DM of substrate, in vitro CH_4_ and NH_3_N reduced by 12% and 47%, respectively; however, the proportion of total volatile fatty acids (VFAs) decreased (−6%) compared to the control diet [9]. Similarly, reductions in enteric CH_4_ (−29%) and urine nitrogen (−9%) were observed when higher amount of mimosa tannin extract was included in the diet of lactating cows, but milk yield dropped by 30% compared with the cows fed the control diet [8]. On the other hand, some studies reported little or no impact of mimosa tannin on rumen fermentation and methane mitigation when added below 50 g/kg DM [10,11]. This is because mimosa tannin is not pure tannin phenolic [12]; it is composed of other non-tannin molecules [13,14,15], in the form of gums, simple sugars, and organic acids [16], which influence the bioactive properties of the tannin, making a lower dosage ineffective. Hence, there is the need for the purification of the mimosa tannin to remove the toxins and other non-tannin substances, thereby reducing the methane yield without compromising the normal rumen function.

Tannin purification is usually achieved with the use of Sephadex LH-20 [17,18,19]. However, the method is very costly and difficult to adopt by most laboratories [16]. Organic solvents, such as ethyl acetate and pentanol, are extensively utilized for extraction processes, coatings, and as carriers in the production of pharmaceuticals, flavorings, inks, adhesives, cosmetics, and antioxidants [20,21,22]. Recently, Missio et al. [16] reported an increase in tannin phenols at the expense of non-tannin compounds when mimosa tannin was purified with ethyl acetate and pentanol, using the simple and less expensive Soxhlet technique. However, to the best of knowledge, there is little or no information on the effect of ethyl acetate purified or pentanol purified mimosa tannin extracts in reducing enteric CH_4_ emission. The objectives of this study, therefore, were to purify the industrially extracted mimosa tannin with ethyl acetate and pentanol using the Soxhlet technique, to characterize the tannin constituents as influenced by the purification with organic solvents, and to evaluate the influence of adding various levels of ethyl acetate and pentanol purified tannin extracts on gas, methane, and in vitro organic matter digestibility of *Eragrostis curvula* hay.

## 2. Materials and Methods

### 2.1. Study Area

This investigation was carried out in the Department of Animal Science, University of Pretoria, South Africa. The area lies at 25°44′30′′ south and 28°15′30′′ east, at an elevation of 1360 m above sea level [23]. The study was reviewed and approved by the Animal Ethics Committee of the University of Pretoria (Ref No: EC075-17).

### 2.2. Materials

The industrially extracted mimosa (*Acacia mearnsii*) tannin powder used in the current study was obtained from UCL Tannin Company Pty (Ltd), Kwa-Zulu Natal, South Africa. The extract was obtained from the bark of a *Acacia mearnsii* tree through a series of hot water extraction processes at a controlled temperature, pressure, and time, followed by vacuum evaporation and subsequent air-drying into fine powder before packaging and storage at −4 °C [16]. The molecular weight of the mimosa ranged from 500–3000 Daltons [24]. The organic solvents used were: ethyl acetate and pentanol, procured from Sigma Aldrich, Johannesburg, South Africa. According to Missio et al. [16], pentanol has a polarity index of 0.568, whereas ethyl acetate has a polarity index of 0.228. Tannic acid, Folin–Ciocalteu reagent, butanol, and all other reagents used were of analytical grades supplied from Sigma Aldrich, Johannesburg, South Africa.

### 2.3. Purification Process

The purification of the mimosa tannin was carried out using the Soxhlet extraction technique as described by Missio et al. [16]. The samples (15 g) of mimosa tannin powder were weighed into thimbles, and fractionated separately with ethyl acetate and pentanol (200 mL) using a Soxhlet apparatus. The purifications were done at the boiling points of the solvents for 6 h (in triplicates). The ethyl acetate and pentanol purified tannin extracts were collected, oven dried at 70 °C, and kept in a refrigerator before analysis.

### 2.4. Tannin Characterization

The powdered samples (200 mg) of unpurified and purified mimosa tannins with ethyl acetate and pentanol were weighed separately in 25 mL volume glass beakers. Aqueous acetone (10 mL) was added and suspended in an ultrasonic bath for 20 min. The contents of the beakers were transferred into the centrifuge tubes, and kept in ice for 15 min. The tubes were then centrifuged at 2500 rpm for 15 min, and the supernatants were collected and kept in ice prior to analysis. The Folin–Ciocalteu method was used to determine the concentrations of total phenols, non-tannin phenols, and total tannins as tannic acid equivalent [25], whereas the proportions of condensed tannins were obtained using the butanol-HCl technique as leucocyanidin equivalent, following the procedure of Porter et al. [26]. The concentration of hydrolysable tannin was calculated by differences between total tannins and condensed tannins according to the method of Singh et al. [27].

### 2.5. Chemical Analysis of Substrate

Freshly harvested *Eragrostis curvula* hay was procured from the Experimental Farm, University of Pretoria, and used as substrate. The sample was milled through a 2 mm screen, and evaluated for chemical composition. Dry matter (DM) and ash were determined according to the method of the Association of Official Analytical Chemists (AOAC) [28]. Nitrogen Analyzer (FP-2000, Leco Instrumente GmbH, Kirchheim, Germany) was used to analyze nitrogen content of the hay, and the value of nitrogen recorded was multiplied by 6.25 to get crude protein (CP). Fibre Analyzer (ANKOM 200/220, ANKOM Technology, New York, NY, USA) was utilized to determine the composition of neutral detergent fiber (NDF), acid detergent fiber (ADF), and acid detergent lignin (ADL) of the substrates in sequence using the procedure of Van Soest et al. [29].

### 2.6. In Vitro Incubation

Buffer was formulated in a three-litres volumetric flask, placed in a water bath, and constantly flushed with saturated carbon di oxide (CO_2_) at 39 °C for about 45 min, as described by Menke and Steingass [30], and modified by Mould et al. [31]. Rumen fluid was collected within the above 45 min period from three rumen fistulated Pinzyl steers fed with *Eragrostis curvula* hay and supplemented with *Medicago sativa* hay. The rumen fluid was squeezed through four folds of cheese cloth, taken to the laboratory without delay in a warmed thermos flask, and continuously flushed with CO_2_. Before incubation, the buffer solution was mixed with the rumen fluid at a ratio of 3:1 into a volumetric flask continuously flushed with CO_2_, and heated at 39 °C in a water bath. The inoculum (40 mL) was then poured into150 mL serum bottles, which already contained 400 mg each of the substrate (*Eragrostis curvula* hay) mixed with 4 mL extracts of unpurified, and purified mimosa tannins with ethyl acetate and pentanol each of them at four different dosages (10, 20, 30, and 40 g/kg DM). The serum bottles were immediately closed tightly using blue rubber stoppers with needles attached to three-way stopcocks, and then incubated at 39 °C, and shook at 120 revolutions per minute (rpm). All the treatments and control were replicated four times, and three blank bottles were included for correction in each incubation run, and four separate runs were conducted in a randomized complete block design (RCBD).

#### 2.6.1. Gas Estimation

The in vitro gas volume produced during the fermentation process was calculated from pressure recorded in per square inch (psi) using a semi-automated pressure Transducer (PX4200-015GI; Omega Engineering Inc., Laval, QC, Canada) connected to a digital data logger (220 series indicators; Omega Engineering Inc.) [32]. The pressures built-up in the serum bottles at 3, 6, 12, 24, and 48 h of incubation were recorded by fitting the transducer into the upper tap of a three-way stopcock connected to the needle with a blue rubber stopper closing the fermentation bottle. The gas readings obtained from the bottles at each period (psi) were corrected by the subtraction of ambient pressure (psi) recorded by the data logger, and then converted to mL using the ANKOM technology equation [33], shown in Equation (1) below:

(1) $$V_{x\;}=V_{j}P_{psi}\;\times \;0.068004084$$

where, *V_x_* = volume of gas in mL, *V_j_* = headspace of serum bottle in mL, and *P_psi_* = corrected pressure recorded in psi by the data logger.

#### 2.6.2. Methane Determination

Methane analysis was performed according to the method of Tavendale et al. [34]. Immediately after reading the pressure built-up in the bottles at 3, 6, 12, 24, and 48 h of incubation periods, gas samples were taken in 10 mL luer-locked syringes, and injected manually into the gas chromatography (SRI GC 8610C BTU Gas Analyzer System, Bad Honnef, Germany) fitted with a flame ionization sensor, and standardized with methane and carbon di oxide following the procedure of Gemeda and Hassen [10]. Methane concentrations were estimated from the area covered by the gas samples in the SRI GC using a standard methane curve.

### 2.7. In Vitro Organic Matter Digestibility Determination (IVOMD)

The IVOMD of *Eragrostis curvula* hay after inclusion of unpurified mimosa tannin, and mimosa tannins purified with ethyl acetate and pentanol at 10, 20, 30, and 40 g/kg DM was assessed using two stage digestion processes, as described by Tilley and Terry [35], with the modifications of Engels and Van der Merwe [36]. Artificial saliva was prepared in a two-litre volumetric flask, kept in a water bath heated at 39 °C, and flushed with saturated CO_2_ continuously. Rumen fluid was obtained from three donor cannulated Pinzyl steers fed with *Eragrostis curvula* hay and supplemented with *Medicago sativa* hay. The rumen fluid was mixed with artificial saliva at a ratio of 1:3, and heated at 39 °C in a water bath with continuous flushing with CO_2_. In stage one, the saliva and rumen fluid mixtures (20 mL) were splashed into digestion tubes, which already contained 200 mg each of the substrate and 2 mL each of urea and tannin extracts (unpurified, ethyl acetate purified, or pentanol purified) at four different concentrations (10, 20, 30, and 40 g/kg DM of feed). The tubes were immediately closed tightly using stoppers fitted with marbles, and digested for 48 h at 39 °C, and shook at 100 rpm. After 48 h, all the tubes were centrifuged at 2500 rpm for 15 min, and the supernatants were carefully removed. In stage two, acid-pepsin solutions (20 mL) were added into the tubes, and digested for another 48 h at 39 °C and 100 rpm. The tubes were removed and centrifuged again at 2500 rpm for 15 min. The supernatants obtained were oven dried at 105 °C for 18 h. The dried supernatants were weighed and ashed at 550 °C in a muffle furnace for three hours. The IVOMD of the substrate was evaluated from the weights of the initial samples, oven dried, and the ash left over. All the treatments and control were in triplicates, three blank tubes were added for correction within each run, and four separate digestion runs were done in RCBD.

### 2.8. Statistical Analysis

All statistical analyses were performed using the general linear model procedure of SAS 9.4 (SAS Institute Inc., Cary, NC, USA). Data on mimosa tannin characterization were analyzed using a one-way ANOVA. Where significant differences existed, means were separated using LSD. Gas production kinetics were evaluated using the Schofield [37] simple exponential Equation (2).

(2) $$V_{t}=v*\left(1-e^{\left(-k*\left(t-l\right)\right)}\right)$$

where *V_t_* = volume of gas at *t* = time; *v* = asymptotic gas volume corresponding to complete substrate digestion; *k* = rate constant; *l* = discrete lag time before gas production commences.

For gas, methane, and in vitro organic matter digestibility, and their ratios and gas kinetics (*v*, *k*, and *l*), the experimental design was a randomized complete block design. The statistical model adopted for the analysis is stated in Equation (3) below:

(3) $$y_{ijk}=\mu +Block+T_{i}+L_{j}+{\left(TL\right)}_{ij\;}+{Ɛ}_{ijk}$$

where *y_ijk_* = observation k for various mimosa tannin extracts, *T* (i; unpurified, ethyl acetate purified and pentanol purified), and level of inclusion, *L* (j; 10, 20, 30, and 40 g/kg DM), of the extracts, *µ* = overall mean, *Block* = blocking effect (incubation runs), *T_i_* = effect of mimosa tannin extract, *L_j_* = effect of inclusion level, *(TL)_ij_* = effect of interaction between tannin extract and inclusion level, and *Ɛ_ijk_* = random error. Significantly different means for unpurified and purified tannin extracts were separated using Tukey’s test. For each tannin extract, single degrees of freedom orthogonal polynomial contrasts (linear, quadratic, and cubic) were used to test the effect of the inclusion level of tannin extracts.

## 3. Results and Discussion

### 3.1. Characterization of Unpurified and Purified Mimosa Tannins

**Figure 1:**
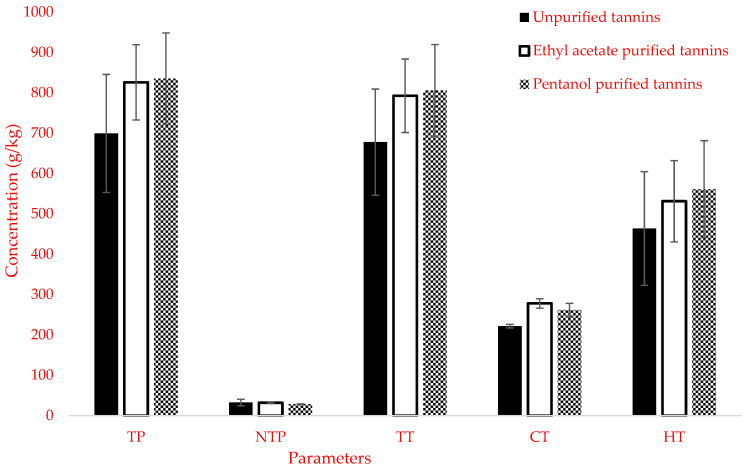
Characterization of unpurified and purified mimosa tannin with ethyl acetate and pentanol. TP = total phenols, NTP = non-tannin phenols, TT = total tannin, CT = condensed tannin, and HT = hydrolysable tannin.

Figure 1 shows tannin characterization for unpurified and purified mimosa tannins. The results indicated that the purification of mimosa tannin with ethyl acetate and pentanol did not influence (*p* > 0.05) the concentration of total phenol (TP), non-tannin phenol (NTP), total tannin (TT), and hydrolysable tannin (HT). However, ethyl acetate purified and pentanol purified extracts had (*p* < 0.05) higher proportion (278.1 g/kg vs. 261.5 g/kg DM) of condensed tannin (CT) compared with purified tannin (221.7 g/kg DM). In general, the Soxhlet purification of the mimosa tannin with organic solvents did not affect any of the parameters measured except condensed tannins, which increased by 26% after purification with ethyl acetate and 18% with pentanol. This suggests that ethyl acetate—being relatively polar solvent, with a lower boiling temperature—has stronger affinity to the CT constituent of the mimosa tannin. The concentration of CT has been shown to decrease with an increase in the polarity of solvents [16]. The proportion of CT recorded in this study for both ethyl acetate purified and pentanol purified mimosa extracts were higher than the 150 g of CT kg^−1^ DM reported by Minho et al. [38], and Bhatta et al. [39], as well as the 235 g/kg DM obtained by Kardel et al. [40], for unpurified mimosa tannin. However, Grainger et al. [8], Carulla et al. [7], and Hassanat and Benchaar [9] reported higher concentrations of CT (603, 615, and 820 g kg^−1^ DM, respectively) after purification of mimosa tannin with a more advanced Sephadex LH-20. Missio et al. [16] suggested that Sephadex LH-20 involves complex analytical procedures and an expensive apparatus; thus, it is difficult to adopt in most laboratories.

### 3.2. Gas, Methane and In Vitro Organic Matter Digestibility

The substrate (*Eragrostis curvula* hay) used in the current study constituted the following: DM (912.1 g/kg), ash (50 g/kg), CP (78.5 g/kg), NDF (698.4 g/kg), ADF (396.1 g/kg), and ADL (59.3 g/kg). The chemical composition showed that the substrate had high fiber and low crude protein, which favors a higher production of hydrogen gas (H_2_), utilized by methanogens to reduce CO_2_ to CH_4_. The current study aimed at examining the effects of unpurified, ethyl acetate purified, and pentanol purified mimosa tannins on suppressing enteric methane production in relation to dry matter digestibility of the substrate.

Table 1 present the summary of gas, methane, and in vitro organic matter digestibility from *Eragrostis curvula* hay after the inclusion of various levels of unpurified, ethyl acetate purified, and pentanol purified mimosa tannin extracts. The results indicated that gas, CH_4_, and IVOMD reduced at a decreasing rate (linear *p* < 0.01) with increasing levels of both purified and non-purified mimosa tannins compared to substrate alone. Compared to control, unpurified and purified tannin extracts reduced gas (*p* < 0.01) at 20–40 g/kg dosages, whereas a significant decrease in CH_4_ was observed only at the 40 g/kg inclusion level. However, the IVOMD of *Eragrostis curvula* hay was affected by all the dosages of both unpurified and purified mimosa tannin extracts. The simple effect of tannin type revealed significant effects of unpurified, ethyl acetate purified, and pentanol purified mimosa tannin extracts on CH_4_ and IVOMD. However, gas volume was not influenced by tannin type (*p* > 0.05). Moreover, inclusion levels reduced gas, CH_4_, and IVOMD (*p* < 0.01) for all the tannin extracts. However, there were no interaction effects (*p* > 0.05) between tannin extracts and inclusion level on gas and CH_4_, whereas IVOMD showed a tendency for tannin extract and dosage interaction.

Generally, the inclusion of 40 g/kg DM of ethyl acetate purified and pentanol purified tannins reduced CH_4_ by approximately 23% each, whereas unpurified extract reduced CH_4_ by 21% at a similar dosage, with little effect on substrate digestibility. The effect of unpurified and purified tannin extracts recorded in this study could be connected to their interference with the proliferation and activities of methanogens. It has been well documented that condensed tannins reduced enteric methane either directly by hindering the activities of methanogenic bacteria, or indirectly by reducing organic matter digestibility [41,42,43,44,45]. This finding is in agreement with that of Hassanat and Benchaar [9], who reported more reduction in gas and CH_4_ with an increase in the concentration of *A. mearnsii* tannin extracts. Tan et al. [46] also reported that increasing levels of *Leucaena leucocephala* CTs in the range of 20 to 60 g/kg DM decreased gas, CH_4_, and IVOMD.

Ethyl acetate purified and pentanol purified tannin extracts reduced more CH_4_ yield than unpurified tannin extract at a similar inclusion level. This could be attributed to the purification effects, which showed a significant increase in condensed tannin concentration for ethyl acetate purified extract. In corroboration with our finding, Tan et al. [46] reported CH_4_ reduction (−33%) at lower concentrations (20 g kg^−1^ DM) of *L. leucocephala* extracts purified using Sephadex LH-20. However, Hassanat and Benchaar [9] reported 9% and 12% reductions in gas and CH_4_, respectively, for unpurified mimosa extract at 50 g/kg dosage, which are lower than the values obtained in this study for ethyl acetate and pentanol purified tannins at a 40 g/kg DM inclusion level. Similarly, Adejoro et al. [24] obtained about a 20% decrease in gas and 24% decrease in CH_4_ after the inclusion of 42 g/kg DM of mimosa tannin extract, which is similar with the percentage reduction recorded in the present study for ethyl acetate purified tannin at a 40 g/kg DM concentration. Carulla et al. [7] also reported around a 13% decrease in CH_4_ at a 25 g/kg DM inclusion level of unpurified mimosa tannin, which is similar to the proportion obtained for pentanol purified tannin, but below that of ethyl acetate purified extract at 20 g/kg DM.

The ratios of gas, methane, and in vitro organic matter digestibility from *Eragrostis curvula* hay supplemented with different levels of unpurified and purified mimosa tannin extracts is summarized in Table 2. The results showed that increases in dosages of purified and non-purified mimosa tannin extracts decreased CH_4_ per unit gas, CH_4_ per unit IVOMD, and gas per unit IVOMD linearly (*p* > 0.01) compared to substrate alone. When compared with the control, unpurified tannin extract mainly reduced (*p* < 0.01) CH_4_/gas and CH_4_/IVOMD at a 40 g/kg DM concentration, whereas ethyl acetate purified extract had a significant effect on CH_4_/gas and CH_4_/IVOMD at a 30–40 g/kg DM inclusion level, and CH_4_/gas and CH_4_/IVOMD were influenced (*p* < 0.01) by the addition of 20–40 g/kg DM of pentanol purified tannin. For gas/IVOMD, both unpurified and purified mimosa tannins had significant effects at 20–40 g/kg DM dosages compared with the control.

The main effect of tannin extract revealed a significant effect of unpurified and purified mimosa tannin extracts on CH_4_/gas and CH_4_/IVOMD. However, gas/IVOMD was not affected (*p* > 0.05) by the purification. Furthermore, a simple effect of tannin concentrations showed that levels of inclusion influenced CH_4_/gas, CH_4_/IVOMD, and gas/IVOMD significantly. There was no significant interaction effect between mimosa tannin type and level of inclusion for CH_4_ per unit gas, CH_4_ per unit IVOMD, and gas per unit IVOMD of the substrate.

In general, the volume of CH_4_ per unit gas, CH_4_ per unit IVOMD, and gas per unit IVOMD decreased with the increase in the concentrations of unpurified and purifed tannin extracts. Moreover, similar inclusion levels of ethyl acetate purified and pentanol purified mimosa extracts had lower CH_4_/gas, CH_4_/IVOMD, and gas/IVOMD compared to unpurified tannin. Pentanol purified tannin was more effective at lower dosage in terms of CH_4_/gas, CH_4_/IVOMD. This suggested that the anti-methanogenic effect of purified tannin extracts was more prominent than their relative effect on organic matter digestibility. This could be linked to the relative increases in CT concentrations due to the purification effects. Condensed tannins have been shown to slow down the activities of microorganisms, as well as the rate of fiber and organic matter degradation, which, in turn, reduces the volume of hydrogen gas (H_2_) required by methanogens to produce CH_4_ [2]. In concurrence with our results, previous in vitro studies also reported a significant reduction in CH_4_/gas, CH_4_/IVOMD, and gas/IVOMD when some tannin rich browse plants [10], and medicinal plant extracts were incubated together with *Eragrostis curvula* hay [47,48].

Table 3 presents the effect of various inclusion levels of unpurified, ethyl acetate purified, and pentanol purified mimosa tannin extracts on gas production indices. The results revealed that the asymptotic gas volume (v) and rate of gas production (k) reduced at a decreasing rate (linear, *p* > 0.01) with increasing levels of unpurified, ethyl acetate purified, and pentanol purified mimosa tannin extracts compared to control. However, compared with control, the lag term (l) was not affected by all the mimosa tannin inclusion levels. Compared with control, though the inclusion of unpurified tannin resulted in lower ‘v’ and ‘k’ at a 40 g/kg dosage, ethyl acetate purified and pentanol purified extract affected ‘v’ and ‘k’ at 30–40 g/kg levels. The simple effect of tannin extracts showed a significant effect of purification on ‘v’. However, ‘k’ and ‘l’ were not affected by tannin types (*p* > 0.05). Likewise, inclusion levels reduced ‘v’ and ‘k’ (*p* < 0.01) with the exception of ‘l’. Though there was no interaction effect (*p* > 0.05) between tannin extracts and dosages on ‘k’ and ‘l’, mimosa extracts and inclusion levels interaction affected ‘v’ significantly.

Generally, potential gas production, as a result of complete substrate digestion and rate of gas production, decreased with the increase in the concentrations of mimosa tannin extracts. Moreover, a similar concentration of purified tannins resulted in lower ‘v’ and ‘k’ compared to the unpurified tannin extract.

Lower asymptotic gas production from in vitro fermentation is linked to a slow rate of organic matter degradation in the rumen [49]. A slow rate of gas production has been attributed to the presence of hydrolysable tannins in mimosa extract, which are believed to be poisonous to rumen microorganisms [50]. However, the anti-methanogenic effect of tannins involves a mixture of direct toxicity on the methanogens, or an indirect reduction in fiber and organic matter degradation [42,50]. The asymptotic gas volume (mL/g) and rate of gas production (h^−1^) obtained in the current study were lower than the 20.2 mL vs. 24.7 mL, and 10.9% vs. 12% per 100 mg DM, reported by Schofield and Pell [51], and Mir et al. [52], for alfalfa and fenugreek, respectively. In addition, longer lag time was recorded by Mir et al. [52] compared to the values obtained in this study. These could be attributed to the variation in chemical composition, particularly the differences in fiber and protein content.

## 4. Conclusions

The findings of this study showed that the Soxhlet purification of mimosa tannin with ethyl acetate and pentanol increased the condensed tannin concentration. However, the highest increase in condensed tannin was achieved by using ethyl acetate. Gas, methane, and in vitro organic matter digestibility of *Eragrostis curvula* hay, and their ratios, decreased with the increase in the inclusion levels of unpurified, ethyl acetate purified, and pentanol purified mimosa tannin extracts. Pentanol purified tannin was more effective at lower dosage in terms of CH_4_/gas, CH_4_/IVOMD. Similarly, asymptotic gas volume and rate of gas production reduced with the increase in the concentration of tannin extracts. Ethyl acetate and pentanol purified mimosa extracts reduced gas and methane volume at a lower dosage (30 g/kg DM) compared to unpurified tannin, which has similar efficacy at a higher level (40 g/kg). It was concluded that the purification of mimosa tannin with ethyl acetate and pentanol reduced potential gas production and CH_4_ with little impact on digestibility when up to 30 g/kg DM of feed was used. Lower inclusion levels of ethyl acetate purified and pentanol purified extracts could give the same effect with higher concentrations of unpurified tannin extract.

## Figures and Tables

**Table 1:** Gas, methane (CH_4_) and in vitro organic matter digestibility (IVOMD) from *Eragrostis curvula* hay incubated with various levels of unpurified, ethyl acetate purified, and pentanol purified mimosa tannin extracts.

Tannin Extracts		Parameters		
	Level (g/kg DM)	Gas (mL/g DM)	CH_4_ (mL/g DM)	IVOMD (g/kg)
Unpurified Tannin	0	155.2 ^a^	7.9 ^a^	600.7 ^a^
	10	149.8 ^ab^	7.4 ^ab^	592.5 ^bc^
	20	145.0 ^bc^	7.1 ^ab^	592.9 ^b^
	30	142.5 ^c^	6.8 ^ab^	589.0 ^bc^
	40	138.2 ^c^	6.2 ^b^	582.5 ^def^
	SEM	1.59	0.33	14.50
	Linear	<0.0.01	0.01	<0.01
	Quadratic	0.45	0.95	0.99
	Cubic	0.65	0.65	0.81
Ethyl acetate purified Tannin	0	155.2 ^a^	7.9 ^a^	600.7 ^a^
	10	150.1 ^ab^	7.3 ^ab^	592.9 ^b^
	20	144.5 ^bc^	6.9 ^ab^	584.7 ^cde^
	30	139.7 ^cd^	6.3 ^ab^	582.4 ^def^
	40	137.3 ^d^	6.1 ^b^	576.3 ^f^
	SEM	1.63	0.41	13.73
	Linear	<0.01	0.01	<0.01
	Quadratic	0.33	0.73	0.86
	Cubic	0.58	0.94	0.94
Pentanol purified Tannin	0	155.2 ^a^	7.9 ^a^	600.7 ^a^
	10	149.4 ^ab^	7.1 ^ab^	592.5 ^bc^
	20	143.7 ^bc^	6.6 ^ab^	585.4 ^cde^
	30	141.5 ^c^	6.40 ^ab^	582.0 ^def^
	40	139.6 ^c^	6.1 ^b^	578.9 ^ef^
	SEM	1.43	0.39	13.84
	Linear	<0.0.01	0.01	<0.01
	Quadratic	0.05	0.42	0.79
	Cubic	0.96	0.77	0.98
SEM		0.414	0.387	0.696
*p* values				
T		0.25	<0.01	<0.01
L		<0.01	<0.01	<0.01
T * L		0.25	0.10	0.05

For each tannin extract means with different superscripts (^a^, ^b^, ^c^, ^d^, ^e^, ^f^) within a column differ significantly at *p <* 0.05. SEM = standard error of mean, T = tannin extract, L= level of inclusion, T * L = interaction effect between tannin extracts and inclusion level.

**Table 2:** The ratios of gas, CH_4_, and IVOMD from *Eragrostis curvula* hay incubated with various levels of unpurified, ethyl acetate purified, and pentanol purified mimosa tannin extracts.

Tannin Extracts		Parameters		
	Level (g/kg DM)	CH_4_/Gas	CH_4_/IVOMD (mL/kg)	Gas/IVOMD (mL/kg)
Unpurified tannin	0	0.051 ^a^	0.013 ^a^	0.259 ^a^
	10	0.050 ^ab^	0.013 ^a^	0.254 ^ab^
	20	0.049 ^abc^	0.012 ^ab^	0.245 ^b–e^
	30	0.048 ^a–d^	0.012 ^ab^	0.243 ^de^
	40	0.045 ^de^	0.011 ^b^	0.238 ^e^
	SEM	0.002	0.005	0.008
	Linear	<0.01	0.003	<0.01
	Quadratic	0.724	0.793	0.832
	Cubic	0.732	0.877	0.969
Ethyl acetate purified tannin	0	0.051 ^a^	0.013 ^a^	0.259 ^a^
	10	0.049 ^abc^	0.012 ^ab^	0.254 ^ab^
	20	0.048 ^a–d^	0.012 ^ab^	0.248 ^bcd^
	30	0.045 ^de^	0.011 ^b^	0.240 ^de^
	40	0.044 ^e^	0.011 ^b^	0.239 ^e^
	SEM	0.003	0.001	0.008
	Linear	<0.01	0.010	<0.01
	Quadratic	0.959	0.837	0.864
	Cubic	0.951	0.808	0.784
Pentanol purified tannin	0	0.051 ^a^	0.013 ^a^	0.259 ^a^
	10	0.048 ^a–d^	0.012 ^ab^	0.253 ^abc^
	20	0.046 ^cde^	0.011 ^bc^	0.246 ^b–e^
	30	0.045 ^de^	0.011 ^bc^	0.244 ^cde^
	40	0.044 ^e^	0.010 ^c^	0.242 ^e^
	SEM	0.003	0.001	0.008
	Linear	<0.01	0.007	<0.01
	Quadratic	0.669	0.688	0.700
	Cubic	0.822	0.721	0.955
SEM		0.0003	0.0001	0.0008
*p*-values				
T		<0.01	0.01	0.48
L		<0.01	<0.01	<0.01
T * L2		0.59	0.40	0.46

For eaqch tannin extract means with different superscripts (^a^, ^b^, ^c^, ^d^, ^e^) within a column differ significantly at *p <* 0.05. SEM = standard error of mean, T = tannin extract, L= level of inclusion, T * L = interaction effect between tannin extracts and inclusion level.

**Table 3:** The gas production kinetics from *Eragrostis curvula* hay incubated with various levels of unpurified, ethyl acetate purified, and pentanol purified mimosa tannin extracts.

Tannin Extracts		Parameters		
	Level (g/kg DM)	v (mL/g DM)	k (mL/h)	l (h)
Unpurified tannin	0	195.7 ^a^	0.034 ^a^	0.017
	10	193.6 ^a^	0.032 ^ab^	0.079
	20	188.6 ^ab^	0.031 ^ab^	0.221
	30	187.9 ^ab^	0.031 ^ab^	0.249
	40	180.7 ^b^	0.030 ^b^	0.086
	SEM	3.566	0.002	0.171
	Linear	0.007	0.022	0.578
	Quadratic	0.659	0.495	0.394
	Cubic	0.749	1.000	0.626
Ethyl acetate purified tannin	0	195.7 ^a^	0.034 ^a^	0.017
	10	192.5 ^ab^	0.032 ^ab^	0.150
	20	186.7 ^ab^	0.031 ^ab^	0.238
	30	182.7 ^b^	0.030 ^b^	0.177
	40	180.4 ^b^	0.030 ^b^	0.144
	SEM	2.881	0.001	0.158
	Linear	0.001	0.02	0.585
	Quadratic	0.748	0.640	0.429
	Cubic	0.636	0.911	0.885
Pentanol purified tannin	0	195.7 ^a^	0.034 ^a^	0.017
	10	192.1 ^ab^	0.032 ^ab^	0.255
	20	187.6 ^ab^	0.031 ^ab^	0.132
	30	187.4 ^b^	0.030 ^b^	0.087
	40	186.7 ^b^	0.029 ^b^	0.083
	SEM	2.700	0.001	0.191
	Linear	0.024	0.022	0.551
	Quadratic	0.220	0.813	0.318
	Cubic	0.769	0.779	0.701
SEM		0.703	0.0003	0.039
*p*-values				
T		0.02	0.43	0.32
L		<0.01	0.01	0.11
T * L		0.03	0.73	0.26

For each tannin extract means with different superscripts (^a^, ^b^) within a column differ significantly at *p <* 0.05. SEM = standard error of mean, T = tannin extract, L = level of inclusion, T * L = interaction effect between tannin extracts and inclusion level, v = asymptotic gas volume, k = rate constant, l = discrete lag term.

## Data Availability

Data contained within the article are available on request from the authors.
